# History of American Medical Association

**Published:** 1855-09

**Authors:** 


*History of the American Medical Association.* By N. S. Davis, M. D., &c., &c., Chicago, Illinois. Edited by S. W. Butler, M. D. Philadelphia. Lippincott, Gambo & Co.

This little volume, of near 200 pages, is certainly a very welcome addition to the other valuable contributions of which we are in the daily receipt. The history of the origin and progress of the American Medical Association is, even now in its infant years, a most interesting record, but in future time when the profession shall have changed, and those hereafter to compose that body, desire to look back upon the circumstances which gave rise to it, as well as those attending its early progress and its doings, will find this a most valuable as well as reliable document. We commend our distinguished northern neighbor for the motive which dictated this offering, and the able manner in which he has executed it. It was fitting and proper that *he* should, while all the facts were fresh and green in his memory, throw them together in form, for the time is coming when the influences of this organization will be more profoundly felt and acknowledged by the profession, and when its powers for good will be more plainly manifest.

The association has numbered but a few years, comparatively in its history, and therefore cannot, nor ought not, to be regarded as more than a bare commencement. Several improvements, however, we think ought to be made in its workings, which we hope will be effected. One very important feature in its organization should be the discussion and settlement of important questions in medicine. This we suppose was the original design, for the very title of the organization would indicate as much. The American Medical Association should establish an American Medical literature, and it is incumbent upon American physicians to do it in an aggregate capacity. Then we should have all the contributions, reports, &c., read before the society, and not send them to a committee of a few, to accept or reject as they in their discretion or judgment may deem best. Is there a member of that body who would be willing to leave the Medical literature of our country to a committee of two or three of his fellow members? Let the Association settle certain disputed questions, and let the majority decide upon them according to the proofs, their experience, and judgment. This is a work of grave import, and time should be given to these weighty considerations. When members will tear away from their cares and duties, it should be done for "no light or transient causes," but from motives growing out of the best interests of science and humanity. When this is the spirit and design of the organization, why hurry away without accomplishing any thing definite? Let them remain together until vexed questions are settled. These objections must be met by a reform, otherwise there will be louder "croaking," more " grumbling," and multiplied " complainings." Let these things be considered, and if our session is to be prolonged, let it be agreed upon and understood. Let us hear from our brethren on the subject.

